# A Sense of Embodiment Is Reflected in People's Signature Size

**DOI:** 10.1371/journal.pone.0088438

**Published:** 2014-02-12

**Authors:** Adhip Rawal, Catherine J. Harmer, Rebecca J. Park, Ursula D. O'Sullivan, J. Mark G. Williams

**Affiliations:** 1 University of Sussex, School of Psychology, Brighton, United Kingdom; 2 University of Oxford, Department of Psychiatry, Warneford Hospital, Oxford, United Kingdom; 3 Southern District Health Board, Dunedin, New Zealand; University of Bologna, Italy

## Abstract

**Background:**

The size of a person's signature may reveal implicit information about how the self is perceived although this has not been closely examined.

**Methods/Results:**

We conducted three experiments to test whether increases in signature size can be induced. Specifically, the aim of these experiments was to test whether changes in signature size reflect a person's current implicit sense of embodiment. Experiment 1 showed that an implicit affect task (positive subliminal evaluative conditioning) led to increases in signature size relative to an affectively neutral task, showing that implicit affective cues alter signature size. Experiments 2 and 3 demonstrated increases in signature size following experiential self-focus on sensory and affective stimuli relative to both conceptual self-focus and external (non-self-focus) in both healthy participants and patients with anorexia nervosa, a disorder associated with self-evaluation and a sense of disembodiment. In all three experiments, increases in signature size were unrelated to changes in self-reported mood and larger than manipulation unrelated variations.

**Conclusions:**

Together, these findings suggest that a person's sense of embodiment is reflected in their signature size.

## Introduction

Variations in the size of a person's signature have long been of interest to graphologists and personality researchers who regard signatures as expressive movements that reveal implicit information about how individuals perceive the self [1]. Few scientific studies have examined this idea with the exception of those by Zweigenhaft and colleagues in the 1970s. This work suggests that differences in signature size reflect variations in perceived social status or self-esteem. Zweigenhaft [2] noticed that U.S. professors had larger signatures than students, observations that were subsequently replicated in U.S. [3] and Irani samples [4] where differences in signature size were apparent even when groups were matched on the size and number of letters that the signatures contained. Furthermore, Zweigenhaft and Marlowe [5] showed that differences in signature size could be induced by experimental manipulations of self-esteem. Students who received positive feedback on a bogus intelligence test or were asked to imagine themselves in powerful roles (e.g., the U.S. president) had larger signatures than age-matched peers who received negative feedback or imagined themselves in less powerful roles (e.g., an office clerk).

These results suggest that processes related to how individuals currently perceive the self influence their signature size. More specifically, Zweigenhaft [6] speculated that the larger signatures in the above studies were due to a momentary *sense* of accomplishment, drawing attention to the experience of physical states that accompanies cognition. Although this idea has not been further examined, it finds support in models of embodied cognition which propose that cognition is grounded in its physical context [7], [8]. According to this view, *experiences* of bodily states are not just sequelae of cognition but fundamental to the operation of cognitive processes. A growing body of evidence supports this proposition. For instance, studies have found that participants' evaluation of stimuli is influenced by subtle manipulations of their postures or facial expressions. Strack, Martin and Stepper [9] showed that participants evaluated cartoons more favorably when they adopted facial expressions that facilitated contraction of the facial musculature involved in smiling (e.g., by holding a pencil between their lips) than when this contraction was inhibited. Wells and Petty [10] found that participants reported more positive attitudes towards a message played to them via headphones when they were concurrently nodding (as opposed to shaking) their head. Similarly, postures and haptic sensations that are incidentally imposed by the environment have been shown to modulate the experience of affect [11], the perception of other people and situations [12], and moral behaviors [13].The evidence from these studies therefore supports the view that physical states play an important role in the construction of mental concepts and that bodily processes are an integral part of cognition. Thus, consistent with Zweigenhaft's [6] reasoning, the experience of bodily processes may account for changes in signature size.

The aim of the present studies was to investigate this embodiment view of signature size. We carried out three experiments to examine this idea. Our first experiment examined whether changes in signature size could be induced by exposing participants to affective stimuli that they are unaware of. Although signature size has been regarded as a measure of implicit processes, this has not been conclusively demonstrated. Effects of implicit affective cues (i.e., those that do not elicit conscious feelings of positive or negative emotional arousal) on cognition have been widely documented [14], suggesting that implicit stimuli may also affect signature size. The effects of bodily states such as in the studies cited above are often implicit which is consistent with the idea that awareness is not a necessary part of the embodiment process [8], [15] and that implicit stimuli may affect signature size when they are associated with the experience of bodily states. Indeed, Duguid and Goncalo [16] recently found that inducing a sense of power caused participants to perceive themselves as taller than their actual height (i.e., altering their perceived body size) in the absence of concurrent changes in verbal reports of mood. Thus, our first experiment examined the impact of an implicit affect task on signature size. We hypothesized that implicit positive affective cues would lead to increases in signature size in the absence of concurrent changes in self-reported mood and self-esteem.

As argued above, the embodiment view of signature size suggests that implicit stimuli affect signature size when they are associated with the experience of bodily states. Experiments two and three were therefore aimed to substantiate this embodiment view by examining the crucial idea that a sense (i.e., the experience) of bodily processes is associated with greater changes in signature size than when bodily processes are just present. Consistent with this idea, Haefner [15] found that individual differences in the sensitivity to stimuli originating inside the body (interoceptive awareness) moderated the influence of bodily cues on cognition. Thus, we hypothesized that participants' signature size would increase when processing information in a manner that enhanced focus on the experience of sensory-perceptual material (i.e., experiential self-focus) compared to focus on the conceptual meaning of such material (i.e., conceptual self-focus; Experiments 2 and 3), or when attention was externally oriented (i.e., non-self-focus; Experiment 3). Experiment 3 also included a small sample of patients with anorexia nervosa (AN). AN is a disorder that is commonly associated with self-evaluation, experiential avoidance, and a sense of disembodiment [17], [18]. Experiential self-focus has been found to ameliorate psychopathology in AN presumably through restoring a sense of the body as it actually is rather than as it is ‘thought’ to be [19], [20]. However, the impact of experiential manipulations on signature size has not been examined in this group although clinical observations have reported small signatures in AN [21], [22]. AN may lend itself particularly well for testing the principle that promoting a sense of embodiment is associated with increases in signature size as this group commonly shows lowered levels of accuracy in body awareness. Thus, Experiment 3 also examined whether experiential self-focus was associated with larger increases in signature size in patients with AN in comparison with healthy controls.

In all experiments, we focussed on within-subject variations in signature size given potential concerns over controlling for stylistic elements of handwriting (e.g., the configuration or shape of letters). Signature size was measured following the procedure by Zweigenhaft and Marlowe [5] (see Experiment 1 for details). We examined the reliability of this procedure by collecting signatures from 30 university students (none of these students participated in the experiments reported in this paper). Signature size measurements were carried out separately by the first author and two independent raters who were not involved in the current experiments or aware of their purpose. The intra-class correlation coefficient (ICC) was .97, suggesting a high degree of measurement consistency across raters. The reliability of this measurement procedure of signature size was verified in Experiment 1 (ICC = .96). For Experiments 2 and 3, measurements were calculated by the first author, following the established procedure.

## Experiment 1

### Materials and Methods

Fifty Oxford University students (36 females, 14 males) free from current or past axis I DSM-IV disorders (as assessed with the Mini-International Neuropsychiatric Interview (MINI) [23]) were randomly assigned to subliminal evaluative (*N* = 23) or non-affective (*N* = 27) conditioning. Prior to the subliminal conditioning procedure, participants completed visual analogue scales (VAS) from 0–10 for current mood and self-esteem (‘happy’, ‘warm towards self’), a state anxiety inventory [24], the Implicit Association Test (IAT) [25] and the Single-Item Self-Esteem Scale (SISE) [26]. Signatures were obtained by asking participants to sign the SISE. There was no restriction of line or space. Signature size reflected the total area covered by the signature (in cm^2^): Height (highest to lowest point)×length (from beginning of the first to the end of the last letter) [5].

**Figure 1:**
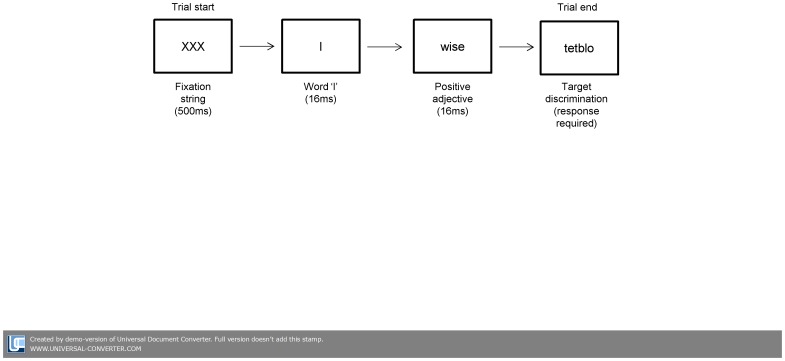
Trial illustration of the subliminal evaluative conditioning procedure. Neutral nouns instead of positive adjectives were presented in the control condition (non-affective conditioning). A presentation rate of 16 ms was chosen as this has been shown to bypass conscious awareness in previous studies.

The subliminal conditioning procedures were identical to those used previously by Dijksterhuis [27] and were presented as part of a target discrimination task that required participants to indicate whether nonsense words began with a vowel or consonant. The task consisted of a total of 30 trials (the interval between trials was 1000 ms). Each trial began with the presentation of a fixation string (‘XXX’) for 500 ms. For participants in the experimental condition (evaluative conditioning), in 15 out of the 30 trials, the fixation string was followed by the presentation of the word ‘I’ and a positive adjective (presented for 16 ms each which has previously been shown to bypass conscious awareness [19]). No subliminal stimuli were presented in the remaining 15 trials. Examples of positive adjectives used in this study were happy, funny, smart and strong (average positive valence rating = 7.59, rating scale from 1–10; [28]). The subliminal stimuli were masked by immediate presentation of a nonsense word. 30 nonsense words were presented (15 beginning with a vowel, 15 beginning with a consonant). At the end of the trial, participants indicated whether the nonsense word began with a vowel or consonant by pressing one of two response keys (see Figure 1 for task procedure). In the control condition (non-affective conditioning) neutral nouns instead of positive adjective were presented (e.g., bench, pillow, paper, and calendar; average positive valence rating = 5.89, rating scale from 1–10; [28]). The procedures were identical otherwise.

Following this manipulation, measurements of mood, self-esteem and signature size were repeated. Participants were then asked whether they were aware of any stimuli that were flashed on screen during the discrimination task after which they completed an awareness task to assess their ability to detect these stimuli. At the beginning of this task, participants were told that they had to pay attention to words that would be flashed on screen following a fixation string. The awareness task consisted of 30 trials: Following presentation of a fixation string (500 ms), a positive adjective or neutral noun (the order was randomized) was presented on screen for 16 ms, which was followed by a nonsense word. Participants were asked to ignore the nonsense word and indicate whether a positive or neutral word had preceded its presentation.

Participants in the two conditions did not differ in terms of the gender ratio, handedness, age, years of education, depression, or anxiety levels (*p*s>.12). All participants signed informed consent prior to taking part in the study. The study was approved by the Oxford University Research Ethics Committee.

### Results and Discussion

A 2 (condition: experimental, control)×2 (time: pre, post)×2 (gender: male, female) repeated measures analysis of variance (ANOVA) indicated a significant increase in signature size following subliminal evaluative relative to non-affective conditioning, *F*(1,46) = 4.03, *p* = .05, partial *η* ^2^ = .08 (Table 1). The average increase in signature size was 13.1% in the experimental condition whereas there was a 1.79% decrease in the control condition. This finding was not moderated by gender (there were no effects of gender throughout the analysis and gender was therefore removed in following analyses). There were no manipulation-related changes on the IAT (a decrease across time, *F*(1,48) = 5.21, *p* = .03, was possibly due to a practice effect) [17], trait, or momentary self-esteem, *F*s<1.78, *p*s>.18. The subliminal conditioning procedure had no effect on mood, *F*s<1.32, *p*s>.25. No participant reported having seen any of the subliminally presented stimuli. Results from the awareness check also indicated no awareness of subliminal stimuli (correct classifications of positive and neutral words was at chance-level: experimental *M* = 14.83 (2.01) vs. control *M* = 14.93 (1.82); *t*(48) = .18, *p* = .86). Thus, subliminal evaluative conditioning induced increases in signature size relative to non-affective conditioning in the absence of changes on subjective reports of mood and self-esteem. These findings substantiate that increases in signature were caused by implicit affective stimuli. There was only a small (non-significant) positive correlation between self-reported trait self-esteem and pre-manipulation signature (*r* = .11, *p* = .44).

**Table 1:** The impact of subliminal evaluative (experimental) and non-affective (control) conditioning on self-esteem, mood and signature size.

	Experimental (*N* = 23)	Control (*N* = 27)
	*M (SD)*	*M (SD)*
IAT		
Pre	.76 (.28)	.79 (.43)
Post	.65 (.27)	.67 (.37)
Happy		
Pre	6.37 (1.53)	6.67 (1.58)
Post	6.47 (1.43)	6.87 (1.43)
State anxiety		
Pre	34.22 (7.21)	31.37 (6.81)
Post	33.39 (8.93)	32.81 (7.73)
Warm towards self		
Pre	6.63 (1.55)	6.70 (1.48)
Post	6.26 (1.74)	6.91 (1.86)
Trait self-esteem		
Pre	4.96 (1.26)	4.96 (1.19)
Post	4.91 (1.24)	5.11 (1.12)
Signature size		
Pre	5.42 (6.48)	6.69 (4.94)
Post	6.13 (5.97)	6.57 (4.48)

*Footnote*. IAT = Implicit Association Test.

These findings suggest that implicit processes contribute to increases in signature size. Prior studies have shown that bodily states have implicit effects on cognition and affect [8], [9], [15]. Thus, one explanation for the effect of implicit affective cues on signature size - consistent with perspectives of embodied cognition - is that this reflects bodily/sensory experiences. If this is the case, increases in signature size should be evident when the experience of bodily processes (i.e., a sense of the body) is facilitated than when it is inhibited. The next two experiments therefore compared the effects of information processing styles that either facilitated (experiential self-focus) or inhibited (conceptual self-focus) experiences of bodily states on signature size and self-report outcomes. This allowed us to further examine an embodiment explanation of signature size.

## Experiment 2

### Materials and Methods

30 female participants (Mean age = 20.53, *SD* = 2.65) free from psychiatric disorder (as determined by the MINI [23]) completed both conceptual and experiential self-focus inductions a week apart from each other in counterbalanced order. Prior to these self-focus inductions, ratings of current mood, self-esteem and signature size were obtained.

The conceptual and experiential self-focus manipulations [29] contain the same 28 items related to self, body-state and emotions (e.g., “the physical sensations in your body”) that participants concentrate upon for eight minutes. The difference between the inductions lies in how to focus attention on this material. The predominant quality of conceptual self-focus is *thinking about* reasons and implications, whereas experiential self-focus is characterized by sustained attention to sensory-perceptual features. The exact directions to participants in the conceptual condition were to “to think about the causes, meanings and consequences of each item and spend a few moments concentrating on each item, attempting to make sense of and understand the issues raised by the item”. Instructions in the experiential condition directed participants “to focus your mind on your experience for each item, concentrating on the quality of what you sense”. In both conditions, participants were asked to read each item silently and slowly to themselves and to work through the list of items at their own pace.

Subsequently, in order to verify induction of the intended style of self-focus, participants were asked to indicate on two VAS (from 0–100) the degree they were processing material (1) conceptually (“I was focussed on trying to understand, explain or make sense of things”) and (2) experientially (“I was focussed on my sensory experience, noticing my body and physical sensations”) during the self-focus tasks. They also completed a third VAS (from 0–100) to verify the overall degree of self-focus during the experimental tasks (“I was focussed on myself”).

Finally, post-manipulation measurements of mood, self-esteem and signature size were obtained. All participants signed informed consent prior to taking part in the study. The study was approved by the Oxford University Research Ethics Committee. Both testing sessions took place in the same setting and at the same time of day whenever possible (i.e., both assessments in the morning or afternoon).

### Results and Discussion

Pre-manipulation signature sizes across conditions were highly correlated, *r* = .90. As in Experiment 1, there was a small (non-significant) positive correlation between self-reported self-esteem and pre-manipulation signature size (*r* = .11, *p* = .58). Both manipulations had similar effects on the extent of self-focus (conceptual *M* = 84.00 (13.92) vs. experiential *M* = 83.50 (20.64); *t*(29) = .14, *p* = .89). The overall degree of self-focus across conditions was not associated with the average change in signature size, *r* = −.17, *p* = .36. The conceptual manipulation led to higher levels of conceptual thinking than the experiential manipulation (*M* = 71.33 (17.86) vs. *M* = 51.83 (28.02); *t*(29) = 3.28, *p*<.01), whereas the opposite pattern was evident on the experiential check (*M* = 54.23 (26.76) vs. *M* = 76.54 (11.44); *t*(29) = 3.85, *p*<.01), suggesting that the manipulation successfully increased focus on sensory-perceptual features.

A repeated measures ANOVA (the factors were time and condition) indicated a significant increase in signature size in the experiential relative to the conceptual self-focus condition, *F*(1,29) = 6.16, *p* = .02, partial *η* ^2^ = .18 (Table 2). The average increase in signature size was 11.41% compared to a 1.71% decrease, consistent with the idea that processing stimuli in a manner that promotes experiential self-focus increases signature size. The self-focus manipulations had no influence on mood or self-esteem, *F*s<2.32, *p*s>.13. The change following experiential self-focus was twice as large compared to manipulation-unrelated change between pre-manipulation measurements (11.41% versus 5.40%).

**Table 2:** The impact of conceptual and experiential self-focus on mood, self-esteem, and signature size.

	Conceptual self-focus	Experiential self-focus
	*M (SD)*	*M (SD)*
Happy		
Pre	6.08 (2.16)	6.88 (1.15)
Post	5.54 (2.29)	6.64 (1.60)
Anxious		
Pre	2.58 (2.33)	2.29 (2.17)
Post	2.84 (2.35)	2.08 (2.24)
At one with self		
Pre	5.98 (2.42)	6.27 (2.27)
Post	6.04 (2.64)	6.60 (2.30)
Signature size		
Pre	3.52 (1.77)	3.33 (1.93)
Post	3.46 (1.81)	3.71 (1.72)

## Experiment 3

### Materials and Methods

Thirteen female Oxford University students and 13 female in-patients with the severe eating disorder AN were recruited. All participants completed three conditions approximately one week apart from each other: conceptual, experiential, and non-self-focus. As in Experiment 2, testing took place in the same setting across the three assessments and at the same time of day whenever possible. The six possible orders of condition were counterbalanced. The two groups were matched on age (*M* = 24.46, *SD* = 4.74 versus *M* = 25.77, *SD* = 4.85; *t*(24) = .70, *p* = .49) and verbal IQ (*M* = 119.77, *SD* = 3.39 versus *M* = 121.00, *SD* = 3.70; *t*(24) = .88, *p* = .39). The procedure was identical to Experiment 2. The only exception was that this experiment included a third (non-self-focus) condition in which participants read facts about DIY (do-it-yourself). The reason for adding this condition was to establish the direction of self-focus effects on signature size. Specifically, this allowed us to examine whether enhancing focus on sensory cues was causally related to increases in signature size, thus supporting the prediction that this was due to a greater sense of embodiment. Although the DIY condition might also facilitate focus on physical experiences (physical actions), this would not necessarily be expected to promote a focus on sensory-perceptual sensations and therefore affect signature size. Ethical approval for the study was obtained and all participants signed informed consent prior to participation.

### Results and Discussion

Pre-manipulation signature sizes across conditions were highly correlated in both groups (healthy group: average *r* = .86; AN: average *r* = .67). The average pre-manipulation signature was smaller in the AN group (*M* = 3.73 (1.75) vs. *M* = 4.39 (1.91)), consistent with earlier reports [21], [22], but this difference was not significant, *t* = .92, *p* = .37, likely due to insufficient power.

Our main interest lay in examining the within-subject effect of the self-focus manipulations. Results from the manipulation checks replicated the pattern from Experiment 2: Higher levels of conceptual thinking were shown following the conceptual versus experiential self-focus induction, *F*(2,23) = 6.89, *p* = .01, whereas higher levels of sensory focus were found in the experiential versus the conceptual self-focus condition, *F*(2,23) = 22.51, *p*<.01 (higher levels of sensory focus were also evident following experiential self-focus compared to the non-self-focus condition). Further, the non-self-focus condition was associated with less self-focus than the other two conditions, *F*(2,23) = 187.20, *p*<.01.

A repeated measures ANOVA showed an increase in signature size following experiential relative to both conceptual and non-self-focus, *F*(2,23) = 4.36, *p* = .03, partial *η* ^2^ = .28 (Table 3), where there was an average increase of 12.50% compared with decreases of 1.29% in the conceptual and 3.38% in the control condition. These effects occurred in the absence of manipulation effects on mood or self-esteem and were not moderated by group, *F*s<1.90, *p*s>.16, although the mean increase in signature size following experiential self-focus was larger in the patient group as we expected (Table 3). Overall, results from this study replicate the findings that increases in signature size reflect the impact of a sense of embodiment.

**Table 3:** The impact of conceptual-, experiential- and non-self-focus on mood, self-esteem, and signature size for healthy participants and patients with anorexia nervosa.

	Conceptual self-focus		Experiential self-focus		Non-self-focus	
	HG (*N* = 13)	AN (*N* = 13)	HG (*N* = 13)	AN (*N* = 13)	HG (*N* = 13)	AN (*N* = 13)
Happy						
Pre	5.89 (1.38)	3.14 (1.55)	6.27 (1.05)	2.18 (1.89)	6.07 (1.21)	3.87 (1.98)
Post	5.28 (1.46)	2.72 (1.70)	5.57 (1.60)	2.23 (2.06)	5.62 (1.03)	4.22 (1.76)
At one with self						
Pre	6.44 (1.53)	2.99 (2.39)	6.72 (1.55)	2.04 (2.28)	6.21 (2.39)	3.08 (2.13)
Post	6.43 (1.75)	2.48 (1.63)	5.82 (2.36)	1.90 (1.65)	6.70 (1.80)	3.56 (2.50)
Signature size						
Pre	4.28 (1.55)	3.46 (1.63)	4.60 (2.49)	3.72 (1.70)	4.28 (1.98)	4.01 (2.57)
Post	4.04 (1.41)	3.61 (2.34)	4.90 (2.41)	4.45 (2.10)	4.37 (1.97)	3.63 (2.07)

*Footnote*: HG = Healthy group; AN = anorexia nervosa. Standard deviations in brackets next to mean values.

## General Discussion

A person's signature size has been said to reveal ‘hidden’ aspects of how the self is perceived although scientific clarification of this claim has been scarce. We conducted three experiments to examine whether changes in signature size can be induced experimentally, specifically by promoting a person's implicit *sense* of embodiment. The processing of physical states is a prerequisite to a felt ‘sense’ of self and the embodied cognition literature suggests that even subtle manipulations of bodily cues (i.e., cues that participants are not aware of) influence cognitive processes [7], [8]. The impact of implicit cues on cognition has been widely demonstrated [14], although no study has examined effects on signature size. Demonstrating that implicit affective cues modulate a person's signature size would thus provide strong support for the view that signature size is related to an implicit sense of self. Indeed, results from our first experiment showed that exposure to implicit positive affective cues led to significant increases in signature size relative to implicit non-affective conditioning. Supporting the view that these effects were due to implicit influences, increases in signature size occurred in the absence of changes in conscious feelings. Further, no participant reported awareness of the subliminal stimuli.

Subsequently, we conducted two further experiments to directly examine the critical claim that increases in signature size are associated with a sense of embodiment. These experiments examined the impact of a focus on the experience of bodily cues relative to conceptually thinking about such material (Experiments 2 and 3) and externally-oriented attention (i.e., non-self-focus; Experiment 3). As hypothesized, results from both experiments showed that experiential processing of sensory and affective material caused within-subject increases in signature size relative to conceptual processing (Experiments 2 and 3) and a non-self-focus control task (Experiment 3). Experiential processing was associated with greater sensory-perceptual focus compared to the other two conditions, supporting the view that a sense of embodiment was causal in increasing signature size. Indeed, the increase in signature size was also evident in relation to the non-self-focus control condition in Experiment 3 which encouraged participants to think about physical actions, thus highlighting that a focus on sensory-perceptual sensations is likely to be the critical dimension underlying increases in signature size, consistent with embodiment views of cognition. Experiment 3 also showed that the same pattern of results was evident in patients with the severe eating disorder AN. This severe eating disorder characteristically features body image distortion, low experiential awareness and a lack of embodiment [30], [31] with anecdotal evidence of small signatures [21], [22]. Aiding experiential self-focus has previously been shown to improve psychopathology in some individuals with AN [19], [20]. The increase in signature size substantiates the view that this is achieved through restoring a more accurate sense of the body as it is. Contrary to our expectation, we did not find significantly greater increases in signature size following experiential self-focus in patients with AN relative to the healthy control group, although the means were in the expected direction. Replication of these findings in larger samples is therefore warranted.

Further, a number of other issues need acknowledgment and require consideration in future studies. First, although changes in signature size were unrelated to explicit assessments of mood and self-esteem this does not rule out that no such effects were present. These may have been obscured by participants' desire to remain consistent in their self-assessment over time when completing mood and self-esteem scales repeatedly in close proximity. Lack of power is an alternative explanation for the absence of concurrent changes in self-reported variables. Second, an important issue that remains to be clarified is whether increases in signature size are specifically related to a positive sense of self as Zweigenhaft [6] speculated. Our studies were not designed to address this issue. However, our findings may suggest that increases in signature size reflect a general sense of embodiment or ‘groundedness’. The items which composed our self-focus manipulations (e.g., ‘the physical sensations in your body’, ‘the way you feel inside’) are not related to positive sensations in an obvious way. Indeed, in patients with AN they may give rise to negative sensations. Nevertheless, an increase in signature size following experiential processing was also apparent in this group. Third, future studies should also rule out the possibility that the increase in signature size following experiential self-focus is related to participants paying more attention to writing their signatures due to the increased focus on sensory-perceptual features (e.g., which may lead them to trying to write more clearly, in turn, possibly inflating signature size). This explanation is unlikely given that Experiment 1 demonstrated increases in signature size following an implicit manipulation, but our studies cannot fully address this.

In conclusion, findings from three experiments provide consistent and convergent support for the suggestion that a person's signature size is associated with an implicit sense of embodiment. As such, measuring changes in signature size provides an unobtrusive method for measuring the impact of subtle cues on cognition.

## References

[1] Allport GW, Vernon PE (1933) Studies in expressive movement. New York: Macmillan.

[2] Zweigenhaft RL (1970) Signature size: A key to status awareness. J Soc Psychol 81: 49–54. 5482079 10.1080/00224545.1970.9919908

[3] Swanson BR, Price RL (1972) Signature size and status. J Soc Psychol 87: 319.

[4] Aiken LR, Zweigenhaft RL (1978) Signature size, sex, and status in Iran. J Soc Psychol 106: 273–274. 28135534 10.1080/00224545.1978.9924179

[5] Zweigenhaft RL, Marlowe D (1973) Signature size: Studies in expressive movement. J Consult Clin Psychol 40: 469–473. 4708124 10.1037/h0034503

[6] Zweigenhaft RL (1977) The empirical study of signature size. Soc Behav Pers 5: 177–185.

[7] Barsalou LW (2008) Grounded cognition. Annu Rev Psychol 59: 617–45. 17705682 10.1146/annurev.psych.59.103006.093639

[8] Niedenthal PM, Barsalou LW, Winkielman P, Krauth-Gruber S, et al (2005) Embodiment in Attitudes, Social Perception, and Emotion. Pers Soc Psychol Rev 9: 184–211. 16083360 10.1207/s15327957pspr0903_1

[9] Strack F, Martin LL, Stepper S (1988) Inhibiting and facilitating conditions of the human smile: A nonobtrusive test of the facial feedback hypothesis. J Per Soc Psychol 54: 768–777. 10.1037//0022-3514.54.5.768 3379579

[10] Wells GL, Petty RE (1980) The effects of overt head movements on persuasion: Compatibility and incompatibility of responses. Bas Appl Psychol 1: 219–230.

[11] Duclos SE, Laird JD, Schneider E, Sexter M, Stern L, et al (1989) Emotion-specific effects of facial expressions and postures on emotional experience. J Per Soc Psychol 57: 100–108.

[12] Ackerman JM, Nocera CC, Bargh JA (2010) Incidental haptic sensations influence social judgments. Science 328: 1712–1715. 20576894 10.1126/science.1189993 PMC3005631

[13] Yap AJ, Wazlawek AS, Lucas BJ, Cuddy AJC, Carney DR (2013) The ergonomics of dishonesty: The effect of incidental posture on stealing, cheating, and traffic violations. Psychol Sci 24: 2281–2289. 24068113 10.1177/0956797613492425

[14] Friedman RS, Förster J (2010) Implicit affective cues and attentional tuning: An integrative review. Psychol Bull 136: 875–893. 20804240 10.1037/a0020495 PMC2933078

[15] Häfner M (2013) When body and mind are talking: Interoception moderates embodied cognition. Exp Psychol 60: 255–259. 23548985 10.1027/1618-3169/a000194

[16] Duguid MM, Goncalo JA (2012) Living large: The powerful overestimate their own height. Psychol Sci 23: 36–40. 22173738 10.1177/0956797611422915

[17] Rawal A, Park RJ, Williams JMG (2010) Rumination, experiential avoidance and dysfunctional thinking in eating disorders. Behav Res Ther 48: 851–859. 20598670 10.1016/j.brat.2010.05.009 PMC2923742

[18] Cowdrey FA, Park RJ (2012) The role of experiential avoidance, rumination and mindfulness in eating disorders. Eat Behav 13: 100–105. 22365790 10.1016/j.eatbeh.2012.01.001

[19] Rawal A, Enayati J, Williams JMG, Park RJ (2009) A mindful approach to eating disorders. Healthcare Counselling and Psychotherapy Journal 9: 16–20.

[20] Rawal A, Williams JMG, Park RJ (2011) Effects of analytical and experiential self-focus on stress-induced cognitive reactivity in eating disorder psychopathology. Behav Res Ther 49: 635–645. 21774916 10.1016/j.brat.2011.06.011 PMC3176901

[21] Beumont P (1971) Small handwriting in some patients with anorexia nervosa. Brit J Psych 119: 349–351. 10.1192/bjp.119.550.349-a 5568206

[22] Sekar MK, Arcelus J, Palmer RL (2010) Micrographia and hypophonia in anorexia nervosa. Int J Eat Disord 43: 762–765. 19885923 10.1002/eat.20768

[23] Sheehan DV, Lecrubier Y, Sheehan K, Amorim P, Janavs J, et al (1998) The development and validation of a structured diagnostic psychiatric interview. J Clin Psychiatry 59: 22–33. 9881538

[24] Spielberger CD, Gorsuch RL, Lushene R, Vagg PR, Jacobs GA (1983) Manual for the state-trait anxiety inventory. Palo Alto, CA: Consulting Psychologists Press.

[25] Greenwald AG, Nosek BA, Banaji MR (2003) Understanding and using the implicit association test: I. An improved scoring algorithm. J Pers Soc Psychol 85: 197–216. 12916565 10.1037/0022-3514.85.2.197

[26] Robins RW, Hendin HM, Trzesniewski KH (2001) Measuring global self-esteem: Construct validation of a single-item measure and the rosenberg self-esteem scale. Pers Soc Psychol Bull 27: 151–161.

[27] Dijksterhuis A (2004) I like myself but I don't know why: Enhancing implicit self-esteem by subliminal evaluative conditioning. J Pers Soc Psychol 86: 345–355. 14769089 10.1037/0022-3514.86.2.345

[28] Warriner AB, Kuperman V, Brybaet M (2013) Norms of valence, arousal, and dominance for 13.915 English lemmas. Behav Res Methods 45: 1191–1207. 23404613 10.3758/s13428-012-0314-x

[29] Watkins E, Teasdale JD (2004) Adaptive and maladaptive self-focus in depression. J Affect Disord 82: 1–8. 15465571 10.1016/j.jad.2003.10.006

[30] Park RJ, Dunn B, Barnard PJ (2011) Schematic models and modes of mind in anorexia nervosa I: A novel process account. Int J Cogn Ther 4: 415–437.

[31] Rosen JC (1990) Body image disturbance in eating disorders. In Cash, TF, Pruzinsky T, editors. Body images: Development, deviance and change: Guilford Press. pp. 190–214.

